# A surrogate FRAX model for Nepal

**DOI:** 10.1007/s11657-024-01474-4

**Published:** 2024-11-15

**Authors:** H. Johansson, D. Pandey, M. Lorentzon, N. C. Harvey, E. V. McCloskey, J. A. Kanis

**Affiliations:** 1 Centre for Metabolic Bone Diseases, University of Sheffield, Sheffield, UK; 2 National Trauma Centre in Katmandu, Katmandu, Nepal; 3 Sahlgrenska Osteoporosis Centre, Institute of Medicine, University of Gothenburg, Gothenburg, Sweden; 4 MRC Lifecourse Epidemiology Centre, University of Southampton, Southampton, UK; 5 NIHR Southampton Biomedical Research Centre, University of Southampton and University Hospital Southampton NHS Foundation Trust, Southampton, UK; 6 Mellanby Centre for Musculoskeletal Research, Division of Clinical Medicine, School of Medicine and Population Health, University of Sheffield, Sheffield, UK

**Keywords:** Nepal, Hip fracture, FRAX, Surrogate model

## Abstract

***Summary*:**

A surrogate FRAX® model for Nepal has been constructed using age- and sex-specific hip fracture rates for Indians living in Singapore and age- and sex-specific mortality rates from Nepal.

**Introduction:**

FRAX models are frequently requested for countries with little or no data on the incidence of hip fractures. In such circumstances, the development of a surrogate FRAX model is recommended based on country-specific mortality data but using fracture data from a country, usually within the region, where fracture rates are considered to be representative of the index country.

**Objective:**

This report describes the development and characteristics of a surrogate FRAX model for Nepal.

**Methods:**

The FRAX model used the ethnic-specific incidence of hip fracture in the Indian community of Singapore, combined with the death risk for Nepal in 2015–2019. The number of hip fractures in 2015 and 2050 was estimated based on the United Nations’ predicted changes in population demography.

**Results:**

The surrogate model gave similar hip fracture probabilities to estimates from Sri Lanka, India and Pakistan but lower 10-year fracture probabilities for men and women at older ages compared to the model for Singapore, reflecting a higher mortality risk in Nepal compared with Singapore. There were very close correlations in fracture probabilities between the Nepalese and the Singapore models (*r* > 0.995) so that the use of the Nepalese model had little impact on the rank order of risk, i.e. a person at the *x*th percentile of risk with one model will be at the *x*th percentile of risk with the other. It was estimated that 6897 hip fractures arose in 2015 in individuals aged 50 years and older in Nepal, with a predicted 3-fold increase expected by 2050, when 23,409 hip fractures are expected nationally.

**Conclusion:**

The surrogate FRAX model for Nepal provides an opportunity to determine fracture probability within the Nepalese population and help guide decisions about treatment.

## Introduction

In 2008, the then WHO Collaborating Centre for Metabolic Bone Diseases at the University of Sheffield, UK, launched the FRAX® tool for the calculation of 10-year fracture probabilities in women and men from readily obtained clinical risk factors (CRFs) with or without bone mineral density (BMD) measurements at the femoral neck (http://www.shef.ac.uk/FRAX). The algorithm (FRAX) was based on a series of meta-analyses using primary data from population-based cohorts that examined a list of candidate clinical risk factors for fracture [1, 2]. The output of FRAX comprises the probability of a major osteoporotic fracture (hip, spine, distal forearm or proximal humerus) or hip fracture. This probability is in turn dependent upon the risk of fracture and the competing risk of death, both of which vary from country to country [3]. Ideally, data for age-specific incidences of fracture and death should be available for the construction of country-specific FRAX models, but information on fracture incidence is frequently poor or absent. On a positive note, the availability of FRAX has stimulated studies of fracture incidence that can be used for the generation of new FRAX models; specific examples include Armenia, Belarus, Brazil, Kazakhstan, Mexico, Moldova, Russia, Turkey and Uzbekistan [4].

Recognising that data on hip and other fractures are not always available, the International Society for Clinical Densitometry and International Osteoporosis Foundation recommend the development of a surrogate FRAX model to be used until country-specific data are collected and made available. Such surrogate models are based on age- and sex-specific mortality data from the index country, combined with age-specific, sex-specific rates of fracture derived from a country, usually nearby, where fracture rates are considered to be representative of the index country [5]. Of the 86 countries for which a FRAX model is available, twelve FRAX country-specific models currently use surrogate data on fracture risk (Bangladesh, Brunei, Ethiopia, Georgia, India, Kyrgystan, Mongolia, Myanmar, Pakistan, Palestine, Sri Lanka and Syria). In the absence of national epidemiological data on fracture [6], the present report describes the development of a surrogate FRAX model for Nepal.

## Methods

The Federal Democratic Republic of Nepal is a landlocked country located in Southern Asia, bordered by the Tibet Autonomous Region of China to the north, and India to the south, east and west. Nepal has an area of 147,516 km^2^ (56,956 sq mi) with a population estimated at 31,235,651 in 2024 [7]. The population of Nepal is young with a median age of 23.7 years against a global value of 30.3 years and a median age of 40.3 years in the UK [8].

### Development of a surrogate model for Nepal

Given its proximity to India, combined with a lack of fracture data from Tibet, it was decided to base the Nepalese model on the fracture rates of the Indian population. Reliable data on the risk of hip fracture from the Indian subcontinent are scarce [6], and data on hip fracture risk were those for the population categorised as of Indian ethnicity in Singapore [9, 10]. Hip fracture rates in Indians living in Singapore are similar to those of Indian ethnicity in South Africa [11, 12]. As described previously, in the absence of incidence data for other sites of major osteoporotic fracture (clinical spine, distal forearm and proximal humerus), the hip fracture rates were used to estimate these incidences on the assumption that the ratio of hip fracture incidence to these other FRAX outcomes is the same in the index country as that documented in Sweden, Iceland, Canada, Moldova and elsewhere [13–16]. National mortality rates for Nepal used data from the World Health Organization for 2015–2019 [17].

### Comparative performance of the surrogate Nepal FRAX model

For the purpose of comparing the authentic FRAX model for Singapore with the surrogate model for Nepal, the probabilities of a major osteoporotic fracture (hip, clinical spine, forearm and humeral fractures) and of hip fracture alone were computed in men and women at ages 50, 60, 70 and 80 years for all possible combinations of FRAX clinical risk factors at BMD *T*-scores between 0 and − 3.5 SD in 0.5 SD steps with a BMI set to 26 kg/m^2^ [18, 19]. This combination of six risk factors and eight values of BMD gave a total of 512 combinations at each age studied. Note that this was not a population simulation, but an array of all possible combinations. The correlation between the probabilities derived from the surrogate and authentic models was examined by linear regression. In a sensitivity analysis, we compared hip fracture probabilities using China as the surrogate country.

The age- and sex-specific incidence was applied to the population of Nepal in 2015 to estimate the number of hip fractures nationwide in that year. Additionally, future projections were estimated up to 2050 assuming that the age- and sex-specific incidence remained stable. Population demography was taken from the United Nations using the medium variant for fertility [7].

## Results

**Fig. 1:**
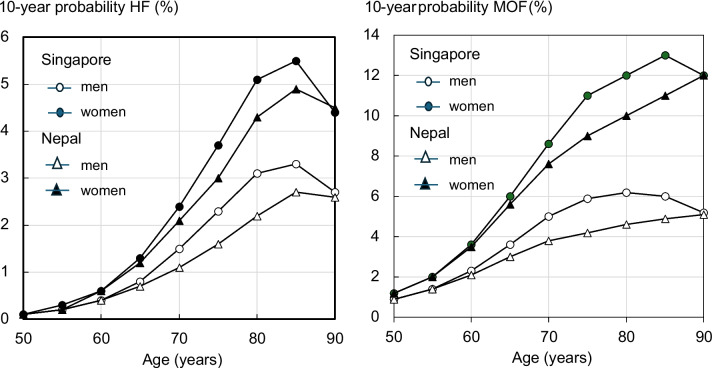
Age-specific 10-year probabilities (%) of a hip fracture (HF) and major osteoporotic fracture (MOF) for men or women without clinical risk factors and BMI of 25 kg/m^2^ with unknown BMD, using Nepali and (Indian) Singaporean FRAX models

The probabilities for Nepal were very similar to those for Singaporean Indians at the age of 50 years, but with increasing age, the difference in median values increased with age, an effect that was more marked for men (Table 1). With advancing age, the surrogate FRAX model gave lower 10-year fracture probabilities for men and women at all older ages except for age 90 years, compared to the model for Indian men and women in Singapore, reflecting a competing higher mortality risk in Nepal (Fig. 1).

**Table 1:** Estimated total number of hip fractures (ICD-10 codes S72.0, S72.1, S72.2) in men and in women aged 50 years and older in 2015 projected up to 2050 in Nepal

	2015	2020	2030	2040	2050
Men	2183	2500	3312	4323	5860
Women	4714	5659	8360	11,885	17,549
Total	6897	8159	11,672	16,208	23,409
Increase (%)	-	118	169	235	339

**Fig. 2:**
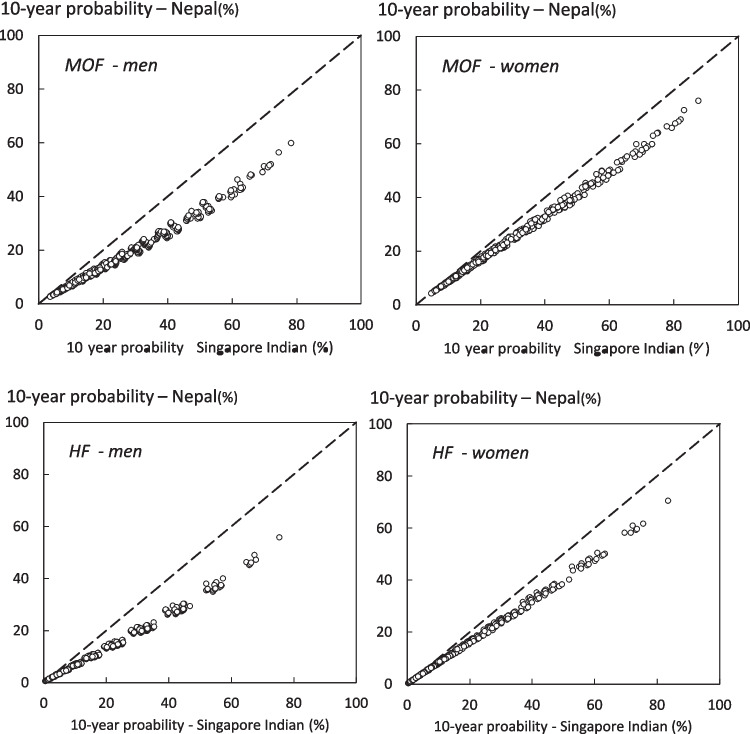
Comparison of 10-year probability of fracture using the surrogate FRAX tool for Nepal and the Singapore Indian FRAX tool for combinations of clinical risk factors and BMD at the age of 70 years. The left-hand panels show the comparison in men. The top panels relate to major osteoporotic fracture (MOF) and the lower panels to hip fracture probability. The diagonal line shows the line of identity

Despite differences in absolute values of probability, there was a close correlation between the FRAX model for Singapore and the surrogate Nepalese model. For all ages the correlation coefficients between the probabilities within risk factor combinations were high (*r* > 0.995). The relationships between the probabilities of a major osteoporotic fracture and hip fracture derived from the two models of FRAX are shown for men and women at age 70 years in Fig. 2.

### Sensitivity analysis

**Fig. 3:**
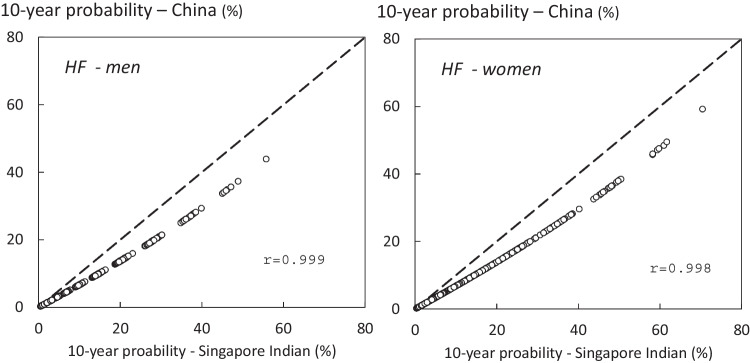
Comparison of 10-year probability of hip fracture (HF) using the surrogate FRAX tool for Nepal derived from Singapore India and mainland China in men and women at the age of 70 years. The diagonal line shows the line of identity

When using mainland China as the surrogate rather than India, probability estimates were 20–25% lower than those derived from the Indian model. As might be expected, there were very close correlations between probabilities from the two models. An example of the comparison is given in Fig. 3 for hip fracture probabilities in men and women at the age of 70 years

### Fracture projections

Assuming that the fracture rates derived from Indians living in Singapore were representative of Nepal and based on the United Nations estimates of the Nepal population for 2015, we estimated that the annual number of hip fractures in men and women aged 50 years or older in Nepal in 2015 totalled 6897, comprising 2183 in men and 4714 fractures in women. The number of hip fractures is estimated to increase progressively by calendar year with a 3-fold increase to 22,768 by 2050 (Table 1).

## Discussion

This paper describes the development of a surrogate FRAX model for Nepal, utilising hip fracture rates in ethnic Indians from Singapore and mortality data from Nepal. With advancing age, the surrogate model provided lower estimates of fracture probability for both major osteoporotic and hip fractures in men and women in Nepal compared with the Singaporean model. The lower probabilities in Nepal reflect differences in age-specific mortality between the two countries. Importantly, the differences had little impact on the stratification of risk, since there was little or no change in the rank order of fracture probability, and the correlation coefficients between surrogate and Singaporean versions were close to unity. Thus, an individual at the 90th percentile of risk in Singapore would still be at the 90th percentile of risk using the surrogate FRAX tool. The lower absolute values of probability would, however, become important in the setting of intervention thresholds and in health economic analysis to inform practice guidelines. For example, the use of thresholds derived from Singapore within Nepal guidelines would have an important impact on the proportion of the population eligible for treatment.

An obvious limitation of this study is the assumption that the fracture rates in Nepal are similar to Indians living in Singapore. This assumption cannot be tested, and differences between the two populations might impact this assumption. It is of interest that hip fracture rates for Indians in Singapore and South Africa are very similar [10, 11] but there are precedents for doubting the adequacy of surrogate models. For example, the historical use of Romanian fracture rates to populate a surrogate Armenian model yielded substantially different probabilities from an authentic model when this became available [20]. In the present study, fracture probabilities were 20–25% lower when mainland China was used as the surrogate. Research is required to derive Nepalese hip fracture incidence data with which to refine this FRAX model.

A further limitation, though one shared with the majority of current FRAX models, is that the model was constructed using incidence data on hip fracture only, rather than all major osteoporotic fractures. The latter are calculated from the hip fracture incidence on the basis that the age- and sex-specific relationship between these fractures and hip fractures is similar to that reported in Malmo, Sweden [13]. Importantly, this commonality of pattern has been observed in other studies where data has allowed its assessment including Canada [14], Iceland [15], the USA [21], the UK [22], Australia [23] and Moldova [16], despite marked differences in incidence between these countries [3]. This commonality of pattern is supported by register studies, which indicate that in those regions where hip fracture rates are high, so too is the risk of forearm fracture and spine fractures (requiring hospital admission) [24, 25].

The widespread availability of FRAX has resulted in its adoption in many practice guidelines worldwide [26]. The fracture probability equivalent to a woman with a prior fracture has been used as an intervention threshold in more than 30 countries [26–30]. If the same threshold were applied to Nepal, then intervention would be recommended with a probability of a major fracture that varied between 2.6 and 19% depending on age. The impact of such thresholds or alternative thresholds will require further study.

In summary, a surrogate FRAX model has been created for Nepal. The model can provide the opportunity to determine fracture probability among the population of Nepal and help guide decisions about treatment. The latter will require the development of assessment and intervention thresholds. Several approaches have been used in practice guidelines worldwide [26], the impact of which will require further study.

## References

[1] Kanis JA on behalf of the World Health Organization Scientific Group (2007) Assessment of osteoporosis at the primary healthcare level. Technical Report. WHO Collaborating Centre, University of Sheffield, UK. Available at http://www.shef.ac.uk/FRAX/index.htm. Accessed 26 Feb 2012

[2] Kanis JA, Johnell O, Oden A, Johansson H, McCloskey EV (2008) FRAX™ and the assessment of fracture probability in men and women from the UK. Osteoporos Int 19:385–397 18292978 10.1007/s00198-007-0543-5 PMC2267485

[3] Kanis JA, Odén A, McCloskey EV, Johansson H, Wahl D, Cyrus Cooper C, on behalf of the IOF Working Group on Epidemiology and Quality of Life (2012) A systematic review of hip fracture incidence and probability of fracture worldwide. Osteoporosis Int 23:2239–2256 10.1007/s00198-012-1964-3 PMC3421108 22419370

[4] Kanis JA, Johansson H, Harvey NC, McCloskey EV (2018) A brief history of FRAX. Arch Osteoporos 13:118. 10.1007/s11657-018-0510-0 30382424 10.1007/s11657-018-0510-0 PMC6290984

[5] Cauley JA, El-Hajj Fuleihan G, Arabi A, Fujiwara S, Ragi-Eis S, Calderon A, Chionh SB, Chen Z, Curtis JR, Danielson ME, Hanley DA, Kroger H, Kung AW, Lesnyak O, Nieves J, Pluskiewicz W, El Rassi R, Silverman S, Schott AM, Rizzoli R, Luckey M, FRAXPosition Conference Members (2011) Official positions for FRAX clinical regarding international differences from Joint Official Positions Development Conference of the International Society for Clinical Densitometry and International Osteoporosis Foundation on FRAX. J Clin Densitom 14(3):240e262 21810532 10.1016/j.jocd.2011.05.015

[6] Dhanwal DK, Siwach R, Dixit V, Mithal A, Jameson K, Cooper C (2013) Incidence of hip fracture in Rohtak district. North India Arch Osteoporos 8:135. 10.1007/s11657-013-0135-2 23620225 10.1007/s11657-013-0135-2 PMC3653238

[7] United Nations (2022) World population prospects 2022. Department of Economic and Social Affairs Population Dynamics. https://population.un.org/wpp/Download/Standard/Population/ Accessed 1 July 2024

[8] Office for National Statistics (2021) Population estimates for the UK, England and Wales, Scotland and Northern Ireland: mid-2019. National and subnational mid-year population estimates for the UK and its constituent countries by administrative area, age and sex. https://www.ons.gov.uk/peoplepopulationandcommunity/populationandmigration/populationestimates/bulletins/annualmidyearpopulationestimates/mid2019estimates#:~:text=The%20average%20age%20in%20England%20reaches%2040%20years%20old&text=In%20mid%2D2019%2C%20the%20median,40.0%20years%20to%2040.1%20years Accessed 19 June 2024

[9] Kanis JA, Chandran M, Chionh SB, Ganeson G, Harvey NC, Koh WP, Kwok T, Lau TC, Liu E, Lorentzon M, McCloskey EV, Tan KB, Vandenput L, Johansson H (2020) Use of age-dependent FRAX-based intervention thresholds for Singapore. Arch Osteoporos 15(1):104. 10.1007/s11657-020-00782-9 32700118 10.1007/s11657-020-00782-9 PMC7376084

[10] Chandran M, McCloskey EV, Thu WPP, Logan S, Hao Y, Tay D, Ang WC, Aung TKK, Choo KS, Ali A, Yan SX, Huang XF, Liu XM, Yong EL, Lekamwasam S (2018) FRAX® based intervention thresholds for management of osteoporosis in Singaporean women. Arch Osteoporos 13:130. 10.1007/s11657-018-0542-5 30456726 10.1007/s11657-018-0542-5

[11] Johansson H, Dela SS, Cassim B, Paruk F, Brown SL, Conradie M, Harvey NC, Jordaan JD, Kalla AA, Liu E, Lorentzon M, Lukhele M, McCloskey EV, Mohamed O, Chutterpaul P, Vandenput L, Kanis JA (2021) FRAX-based fracture probabilities in South Africa. Arch Osteoporos 16(1):51. 10.1007/s11657-021-00905-w 33649966 10.1007/s11657-021-00905-w PMC7921059

[12] Dela SS, Paruk F, Brown SL, Lukhele M, Kalla AA, Jordaan JD, Conradie M, Mohamed O, Chutterpaul P, Cassim B (2020) Ethnic and gender-specific incidence rates for hip fractures in South Africa: a multi-centre study. Bone 133:115253. 10.1016/j.bone.2020.115253 31987987 10.1016/j.bone.2020.115253

[13] Kanis JA, Oden A, Johnell O, Jonsson B, de Laet C, Dawson A (2001) The burden of osteoporotic fractures: a method for setting intervention thresholds. Osteoporos Int 12:417–427 11444092 10.1007/s001980170112

[14] Lam A, LeslieWD LLM, Yogendran M, Morin SN, Majumdar SR (2014) Major osteoporotic to hip fracture ratios in Canadian men and women with Swedish comparisons: a population-based analysis. J Bone Miner Res 29:1067–1073 24243719 10.1002/jbmr.2146

[15] Siggeirsdottir K, Aspelund T, Johansson H, Gudmundsson EF, Mogensen B, Jonsson BY, Gudnason V, McCloskey E, Oden A, Sigurdsson G, Kanis JA (2014) The incidence of a first major osteoporotic fracture in Iceland and implications for FRAX. Osteoporos Int 25:2445–2451 24980183 10.1007/s00198-014-2777-3

[16] Zakroyeva A, Lesnyak O, Cazac V, Groppa L, Russu E, Chislari L, Rotaru L, Johansson H, Harvey NC, McCloskey E, Kanis JA (2020) Epidemiology of osteoporotic fracture in Moldova and development of a country specific FRAX model. Arch Osteoporos 15(1):13. 10.1007/s11657-019-0669-z 31993755 10.1007/s11657-019-0669-z PMC6987067

[17] World Health Organization (2018) Health statistics and information systems: http://www.who.int/healthinfo/statistics/mortality_rawdata/en/ Accessed 17^th^ April 2021

[18] Kanis JA, Johansson H, Oden A, Dawson-Hughes B, Melton LJ 3rd, McCloskey EV (2010) The effects of a FRAX(®) revision for the USA. Osteoporos Int 21:35–40 19705047 10.1007/s00198-009-1033-8

[19] Lesnyak O, Zakroyeva A, Lobanchenko O, Johansson H, Liu E, Lorentzon M, Harvey NC, McCloskey E, Kanis JA (2020) A surrogate FRAX model for the Kyrgyz Republic. Arch Osteoporos 15:68. 10.1007/s11657-020-00743-2 32377964 10.1007/s11657-020-00743-2 PMC7203583

[20] Lesnyak O, Sahakyan S, Zakroyeva A, Bilezikian JP, Hutchings N, Galstyan R, Lebedev A, Johansson H, Harvey NC, McCloskey E, Kanis JA (2017) Epidemiology of fractures in Armenia: development of a country-specific FRAX model and comparison to its surrogate. Arch Osteoporos 12(1):98. 10.1007/s11657-017-0392-6 29116417 10.1007/s11657-017-0392-6 PMC5676826

[21] Melton LJ, Crowson CS, O’Fallon WM (1999) Fracture incidence in Olmsted County, Minnesota: comparison of urban and with rural rates and changes in urban rates over time. Osteoporos Int 9:29–37 10367027 10.1007/s001980050113

[22] Singer BR, McLauchlan CJ, Robinson CM, Christie J (1998) Epidemiology of fracture in 15.000 adults. The influence of age and gender. J Bone Joint Surg 80B:234–238 10.1302/0301-620x.80b2.7762 9546453

[23] Sanders KM, Seeman E, Ugoni AM, Pasco JA, Martin TJ, Skoric B, Nicholson GC, Kotowicz MA (1999) Age- and gender specific rate of fractures in Australia: a population-based study. Osteoporos Int 10(2):40–47 10.1007/s001980050222 10525717

[24] Johnell O, Gullberg B, Kanis JA (1997) The hospital burden of vertebral fracture in Europe: a study of national register sources. Osteoporos Int 7:138–144 9166394 10.1007/BF01623689

[25] Melton LJ (1995) Epidemiology of fractures. In: Riggs BL, Melton LJ (eds) Osteoporosis: etiology, diagnosis and management, 2ndedn. Lippincott-Raven, Philadelphia, pp 225–227

[26] Kanis JA, Harvey NC, Cyrus Cooper C, Johansson H, Odén A, McCloskey EV, the Advisory Board of the National Osteoporosis Guideline Group (2016) A systematic review of intervention thresholds based on FRAX. A report prepared for the National Osteoporosis Guideline Group and the International Osteoporosis Foundation. Arch Osteoporos 11:25. 10.1007/s11657-016-0278-z 10.1007/s11657-016-0278-z PMC4978487 27465509

[27] Kanis JA, Cooper C, Rizzoli R, Reginster J-Y, Scientific Advisory Board of the European Society for Clinical and Economic Aspects of Osteoporosis (ESCEO) and the Committees of Scientific Advisors and National Societies of the International Osteoporosis Foundation (IOF) (2019) European guidance for the diagnosis and management of osteoporosis in postmenopausal women. Osteoporos Int 30:3–44 30324412 10.1007/s00198-018-4704-5 PMC7026233

[28] Lesnyak O, Zakroyeva A, Babalyan V, Cazac V, Gabdulina G, Ismailov S, Lobanchenko O, Rudenka E, Tsagareli M, Johansson H, Harvey NC, McCloskey E, Kanis JA (2021) FRAX-based intervention thresholds in eight Eurasian countries: Armenia, Belarus, Georgia, Kazakhstan, the Kyrgyz Republic, Moldova, the Russian Federation, and Uzbekistan. Arch Osteoporos 16(1):87. 10.1007/s11657-021-00962-1 34089424 10.1007/s11657-021-00962-1

[29] Naseri A, Bakhshayeshkaram M, Salehi S, Heydari ST, Dabbaghmanesh MH (2024) Dabbaghmanesh MM (2024) FRAX-derived intervention and assessment thresholds for osteoporosis in ten Middle Eastern countries. Arch Osteoporos 19(1):41. 10.1007/s11657-024-01397-0 38780743 10.1007/s11657-024-01397-0

[30] Clark P, Denova-Gutiérrez E, Zerbini C, Sanchez A, Messina O, Jaller JJ, Campusano C, Orces CH, Riera G, Johansson H, Kanis JA (2018) FRAX-based intervention and assessment thresholds in seven Latin American countries. Osteoporos Int 29:707–715 29273826 10.1007/s00198-017-4341-4

